# The involvement of GABA-rho receptors in regulating ethanol-induced elevation of dopamine, glycine and taurine within the nucleus accumbens of Wistar rats

**DOI:** 10.3389/fphar.2025.1668669

**Published:** 2025-10-28

**Authors:** Davide Cadeddu, Anna Loftén, Karin Ademar, Bo Söderpalm, Louise Adermark, Mia Ericson

**Affiliations:** ^1^ Department of Pharmacology, Institute of Neuroscience and Physiology, The Sahlgrenska Academy at University of Gothenburg, Gothenburg, Sweden; ^2^ Addiction Biology Unit, Department of Psychiatry and Neurochemistry, Institute of Neuroscience and Physiology, The Sahlgrenska Academy at University of Gothenburg, Gothenburg, Sweden; ^3^ Beroendekliniken, Sahlgrenska University Hospital, Gothenburg, Sweden

**Keywords:** dopamine, ethanol, taurine, GABA-rho, rat, nucleus accumbens, glycine

## Abstract

**Introduction:**

Alcohol use disorder (AUD) causes significant morbidity and mortality globally. Ethanol’s rewarding and reinforcing effects are attributed to activation of the mesolimbic dopamine system, increasing accumbal dopamine release. While activation of accumbal glycine receptors (GlyRs) is a prerequisite for ethanol-induced dopamine signaling, multiple transmitter systems may be involved; recent research implicates the GABA-rho receptor as a prominent target. Considering the structural and functional similarities between GlyRs and GABA-rho receptors, this study aimed to define the role of GlyRs and GABA-rho receptors in regulating baseline dopamine signalling and ethanol-induced elevation of extracellular dopamine and GlyR agonists, as well as to determine their involvement in the action of the ethanol relapse-preventing drug acamprosate.

**Methods:**

To investigate this, *in vivo* microdialysis was conducted in male Wistar rats.

**Results and discussion:**

Local perfusion with either the GABA-rho receptor antagonist TPMPA or the GlyR antagonist strychnine prior to ethanol administration significantly reduced the ethanol-induced increase in dopamine levels. These findings suggest that both GlyRs and GABA-rho receptors are involved in mediating the dopamine-elevating effect of ethanol. In addition, a significant attenuation of the ethanol-induced glycine and taurine elevation was observed following both pretreatment with TPMPA and strychnine, whilst only GlyR blockade inhibited the acamprosate-induced increase of dopamine. Unlike strychnine, TPMPA alone did not alter dopamine levels, suggesting that GABA-rho receptors display features that distinguish them from GlyR. In conclusion, GABA-rho receptors regulate ethanol-induced dopamine and glycine/taurine levels within the nAc without affecting basal dopamine neurotransmission, suggesting their potential as a pharmacological target for the treatment of AUD.

## 1 Introduction

With 3 million deaths per year (5.3% of global deaths), alcohol consumption is one of the main risk factors of premature death worldwide, and it accounts for 5.1% of the global burden of disease (Who, 2018). Despite the high prevalence, treatment options are few and defining new interventions is imperative. Ethanol exerts its effects on the brain by altering the functionality of brain areas involved in the rewarding and reinforcing properties of drugs of abuse. Especially, the rewarding property of ethanol has been attributed to the increase in dopamine release from dopaminergic neurons located in the ventral tegmental area (VTA) projecting to the nucleus accumbens (nAc) (Di Chiara, 1997; Imperato and Di Chiara, 1986). Acamprosate is one of few approved pharmacological treatments for alcohol use disorder (AUD). Our research group, and others, have shown that acamprosate increases dopamine in the mesolimbic dopamine system (Cano-Cebrian et al., 2003; Chau et al., 2010a; Olive et al., 2002), suggesting that acamprosate may act as a partial neurochemical substitution for ethanol (Ademar et al., 2022; Ademar et al., 2023; Chau et al., 2018).

While many neurotransmitter systems may be involved in regulating ethanol-induced dopamine release (Abrahao et al., 2017), inhibition of accumbal glycine receptors (GlyRs) both reduces basal dopamine levels in the nAc (Molander and Söderpalm, 2005a), and prevents ethanol-induced dopamine elevation (Molander and Söderpalm, 2005b). Furthermore, GlyRs may be involved in the alcohol relapse-preventing effect of acamprosate, as antagonism of GlyRs also blocks the acamprosate-induced elevation of dopamine and reverses the alcohol intake reducing effect of the drug in rats (Ademar et al., 2022; Chau et al., 2010a; Chau et al., 2010b). Recently, the atypical GABAergic receptor, GABA-rho (also known as GABA-ρ or GABA_C_ or GABAA_C_), has received attention as a regulator of ethanol-induced dopamine release (Yorgason et al., 2022) and voluntary ethanol consumption (Blednov et al., 2014). Unlike GABA_A_ and GABA_B_ receptors, GABA-rho receptors are insensitive to bicuculline, baclofen, benzodiazepines, neurosteroids, and barbiturates (Naffaa et al., 2017; Woodward et al., 1993). However, they are selectively blocked by the competitive antagonist (1,2,5,6-tetrahydropyridin-4-yl) methylphosphinic acid (TPMPA) (Bormann, 2000; Mohammadi et al., 2017; Zhang et al., 2001) and are sensitive to the GlyR agonists glycine and taurine (Ochoa-de la Paz et al., 2008). This suggests that GABA-rho receptors in certain aspects show more similarities to GlyRs than to GABA-A receptors.

Given the role of GlyRs in regulating ethanol-induced responses and the similarity between GlyRs and the GABA-rho in receptor activation, we hypothesized that GlyR and GABA-rho would display comparable mechanisms of action. To test this hypothesis, we outlined the role of GABA-rho receptors and GlyRs in regulating basal dopamine levels and ethanol-induced dopamine elevation *in vivo*. We further assessed their involvement in ethanol-induced elevation of glycine receptor agonists (glycine and taurine), and in controlling acamprosate-induced dopamine elevation using *in vivo* microdialysis in Wistar rats.

## 2 Materials and methods

### 2.1 Animals

Male Wistar RccHan rats (Inotiv, the Netherlands) weighing 250–280 g (corresponding to an age of 9–10 weeks) at arrival to the facility were group-housed at a constant room temperature of 20–22 °C, humidity of 55–65% and with a regular light/dark cycle (light on at 7:00 a.m./light off at 7:00 p.m.). The rats had free access to regular rat chow and water and were allowed to acclimatize to the new environment for 1 week prior to the initiation of any experiments. All experiments were approved by the Ethics Committee for Animal Experiments, Gothenburg, Sweden (2401/19).

### 2.2 Drugs

(1,2,5,6-tetrahydropyridine-4-yl)methylphosphinic acid (TPMPA) (Tocris and Cayman Chemical Company) (10 μM, 100 μM or 500 μM), strychnine hydrochloride (Sigma-Aldrich, Stockholm, Sweden) (20 μM or 200 μM), calcium-bis(N-acetylhomotaurinate) (acamprosate) (Merck, Lyon, France) (1 mM), ethanol (95% Solveco, Sweden) (300 mM), taurine (50 µM) and glycine (50 µM) (Sigma-Aldrich, Stockholm, Sweden) were used in the study. All drugs were dissolved in Ringer’s solution (140 mM NaCl, 1.2 mM CaCl_2_, 3.0 mM KCl, and 1.0 mM MgCl_2_) and administered locally via reversed microdialysis in the nAc.

### 2.3 Surgery

Rats were deeply anesthetized with 4% isoflurane (Baxter Medical AB, Kista, Sweden), mounted onto a stereotaxic instrument (David Kopf Instruments, Lidingö, Sweden) and placed on a heating pad to prevent hypothermia. The skull was exposed, and three holes were drilled: one above the target area, the nAc, and two for attachment of anchoring screws. An I-shaped custom-made probe was implanted into the nAc core/shell borderline region (A/P: + 1.85, M/L: −1.4 relative to bregma, D/V: −7.8 mm relative to dura mater; Paxinos and Watson 7^th^ compact ed. 2018) and fixed using Harvard cement (DAB Dental AB, Stockholm, Sweden). An active space of 2 mm and a molecular cut-off of 20 kDa was used for the dialysis probe. To prevent dehydration, rats received saline solution (2 mL, NaCl 0.9%) via subcutaneous (s.c.) injection at the end of the surgical procedure. Metacam vet (2 mg/mL, 1 mg/kg, s. c.; Apoteket AB, Sweden) was used as perioperative analgesia. Prior to the *in vivo* microdialysis experiments, rats were single-housed and allowed to recover for 48 h.

### 2.4 *In vivo* microdialysis

**FIGURE 1:**
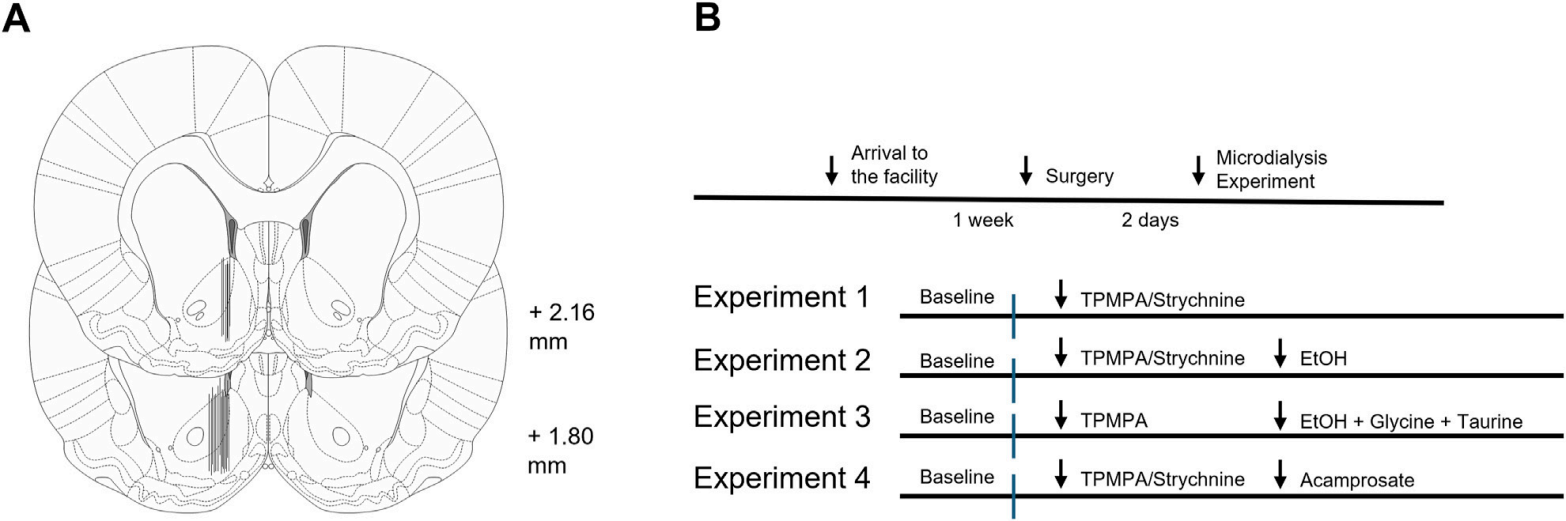
Histology and experimental design. **(A)** Schematic drawing of rat brain slices representing the nucleus accumbens. Black lines represent dialysis probe placements of approximately every fourth animal. Numbers indicate distance from bregma. **(B)** Schematic overview of the experimental design.

On the day of the experiment, rats were allowed to move freely in the cage. The probe was connected, via a swivel, to a microinjection pump (U-864 Syringe Pump, AgnTho’s, Lidingö Sweden), and perfused with Ringer’s solution (rate of 2 μL/min). A 2-h equilibrium phase was performed to stabilize fluid exchange, after which samples (40 μL) were collected every 20 min. A baseline period of 80 min was followed by the drug local administration of the drug when applicable. The microdialysate was analyzed using high-performance liquid chromatography (HPLC) with electrochemical detection to determine DA concentration, as previously described (Clarke et al., 2014). Additionally, HPLC with fluorescence detection was used to analyze levels of amino acids (glycine and taurine) as described previously (Ulenius et al., 2020). At the end of the experiment, rats were euthanized, brains gently removed from the skull and fixed in Accustain (Sigma diagnostics, United States) for 5–7 days. Brains were sectioned using a vibroslicer (Campden Instruments Ltd., Lafayette, IN, United States) to verify correct probe placement by gross examination (Figure 1A). Rats with incorrect probe placement or visual defects were excluded from the statistical analysis.

### 2.5 Experimental design

In a first experiment, TPMPA (10 μM, 100 μM, and 500 μM) or strychnine (20 μM and 200 μM) was administered in the nAc via the microdialysis probe to assess whether GABA-rho and GlyRs both are involved in maintaining basal dopamine levels within the nAc. In a second experiment, TPMPA (10 μM or 100 μM) or strychnine (20 μM) was administered in the nAc via the microdialysis probe prior to ethanol (300 mM, corresponding to approximately 67 mM in the tissue (Loftén et al., 2025)) locally in the nAc to assess if GABA-rho receptors and GlyRs both are involved in the ethanol-induced dopamine elevation. In the second experiment, we also analyzed the GlyR agonists glycine and taurine. In a third experiment we wanted to investigate if the addition of 50 μM glycine and 50 μM taurine to ethanol (300 mM) following TPMPA (100 μM) pretreatment would influence extracellular nAc dopamine. In the last study, we wanted to explore the influence of GABA-rho receptors on acamprosate-induced dopamine elevation. Here we perfused TPMPA (100 μM) or strychnine (20 μM) before the addition of acamprosate (1 mM). All drugs were applied via the dialysis probe. Experimental design is visualized in Figure 1B.

### 2.6 Statistical analysis

GraphPad Prism software version 10 (GraphPad Software, Inc., San Diego, CA, United States) was used for all statistical analyses. Two-way repeated-measures ANOVA followed by Tukey’s *post hoc* analysis was used for statistical evaluation of dopamine, glycine, and taurine levels for relevant timepoints. Only time points after the pharmacological intervention were included in the analysis. All data are presented as mean ± standard error of the mean (SEM), and *p*-value <0.05 was considered statistically significant.

## 3 Results

### 3.1 Glycine receptors, but not GABA-rho receptors, participate in maintaining the nucleus accumbens dopamine tone

**FIGURE 2:**
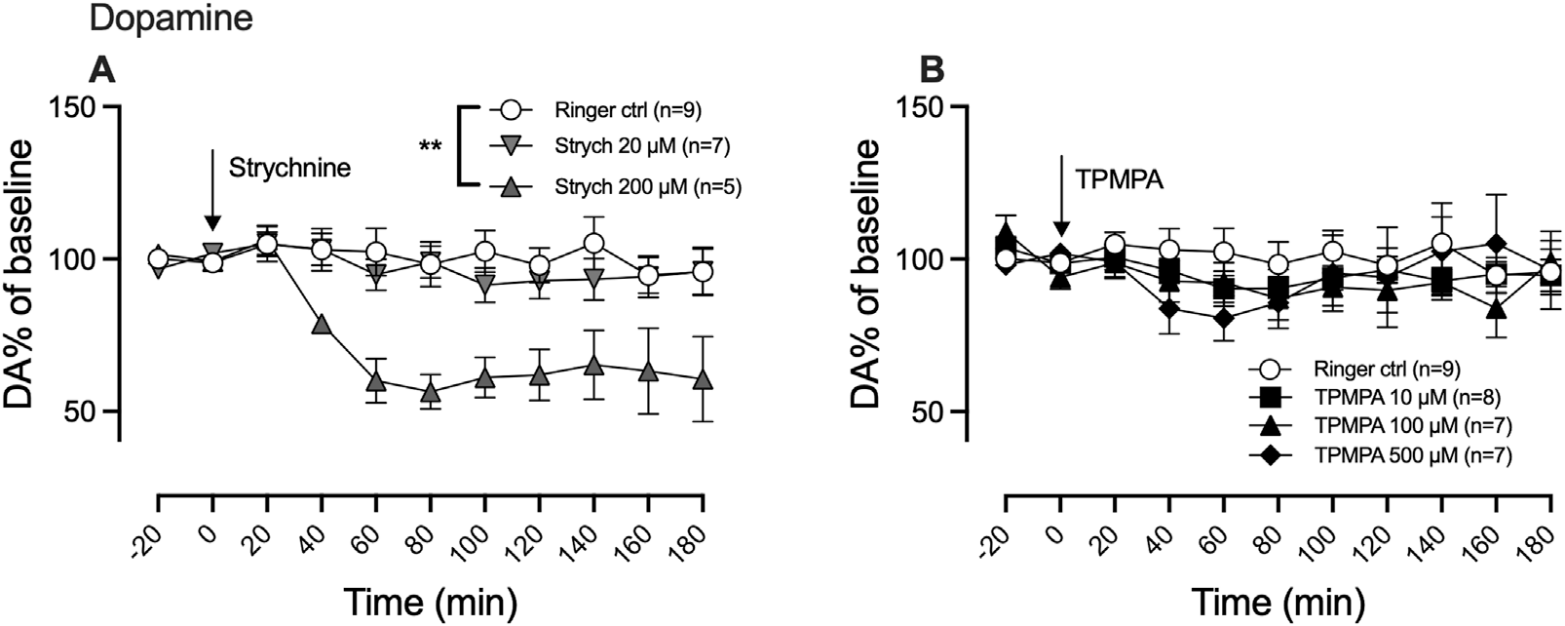
Glycine receptors, but not GABA-rho receptors, participate in maintaining the nucleus accumbens (nAc) dopamine tone. Strychnine (200 μM; Strych) significantly decreased nAc dopamine (DA) as compared to Ringer perfused rats **(A)**, as measured by *in vivo* microdialysis in freely moving Wistar rats. Neither of the perfused doses of TPMPA produced a significant effect on nAc DA **(B)**. Arrows indicate start of drug perfusion. Data are shown as means ± SEM. Statistical significance was determined by two-way repeated measures ANOVA followed by Tukey’s *post hoc* test, ** denotes p < 0.01.

We previously demonstrated that a high concentration of the GlyR antagonist strychnine (200 μM) decreases nAc dopamine levels in a manner that can be reversed by glycine, suggesting that GlyRs are involved in maintaining basal dopamine tone within the nAc. Since the GABA-rho receptor and GlyR share similar traits, we wanted to investigate whether the GABA-rho receptor also could be involved in regulating basal dopamine levels. As previously demonstrated, we found a dose-dependent decrease of nAc dopamine following strychnine administration (two-way ANOVA with repeated measures time points 20–180 min; F (2, 18) = 7.043; p = 0.0054, *post hoc* analysis, Ringer vs. strychnine 20, p = 0.883, Ringer vs. strychnine 200, p = 0.005, strychnine 20 vs. strychnine 200 p = 0.0189) (Figure 2A). Following perfusion of increasing concentrations of TPMPA, we did not find any influence on the extracellular levels of dopamine by the drug alone (two-way ANOVA with repeated measures time points 20–180 min; F (3, 28) = 2.0; p = 0.1263) (Figure 2B).

### 3.2 Ethanol-induced dopamine elevation is blocked by the GABA-rho receptor antagonist TPMPA or the glycine receptor antagonist strychnine

**FIGURE 3:**
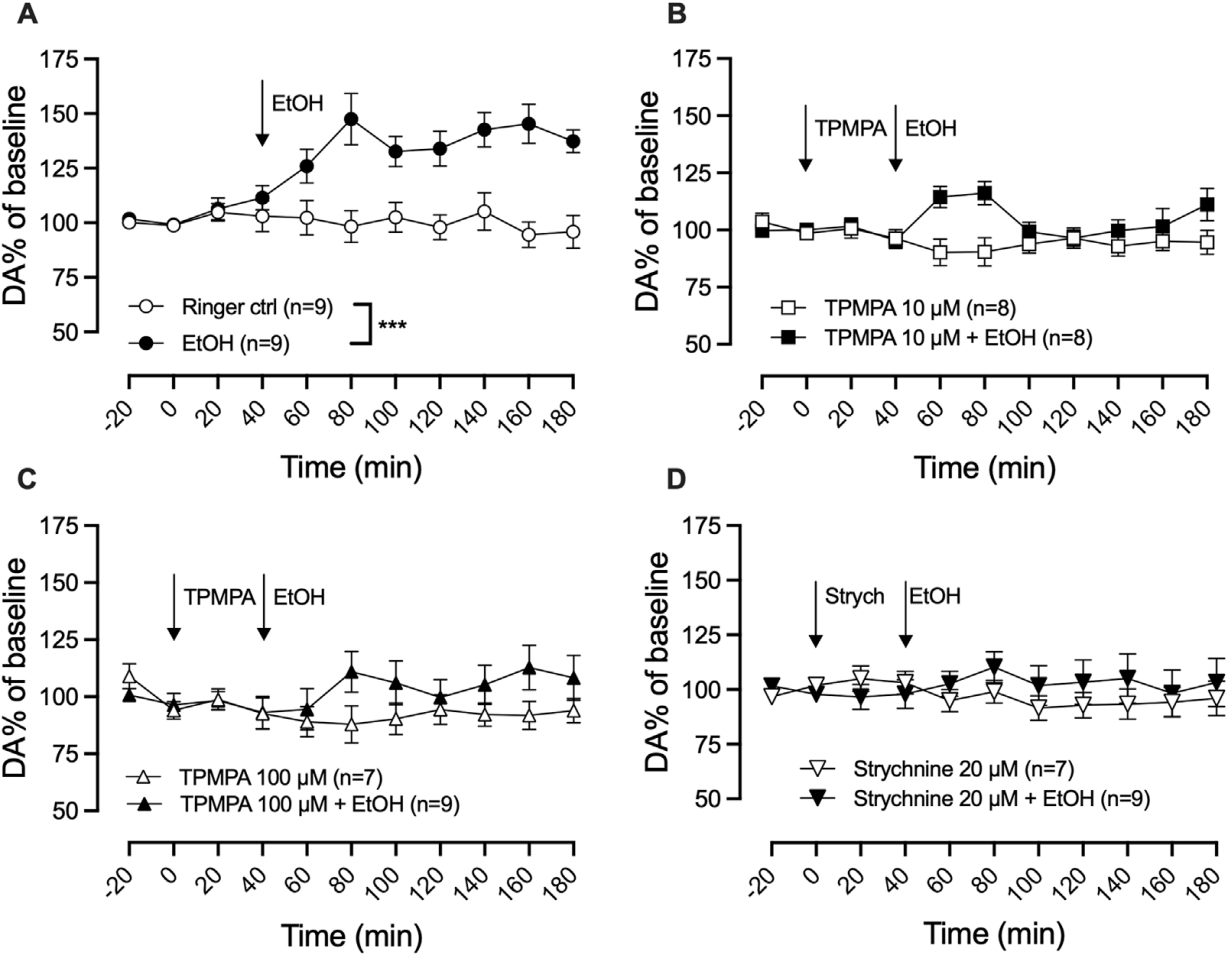
GABA-rho receptor or glycine receptor blockade attenuate ethanol-induced dopamine elevation in the nucleus accumbens. Time-course graph of dopamine (DA) levels as measured by *in vivo* microdialysis in freely moving Wistar rats, presented as DA percent of baseline following local perfusion of Ringer’s solution and ethanol (EtOH) 300 mM **(A)**, TPMPA 10 μM alone and in combination with EtOH 300 mM **(B)**, or TPMPA 100 μM alone and in combination with EtOH 300 mM **(C)**, strychnine (Strych) 20 μM alone or in combination with EtOH 300 mM **(D)**. Arrows indicate start of local drug administration. Data are shown as means ± SEM. Statistical significance was determined by two-way repeated measures ANOVA followed by Tukey’s *post hoc* test, *** denotes p < 0.001.

To explore the involvement of GABA-rho receptors in ethanol-induced dopamine release, ethanol and the GABA-rho receptor antagonist TPMPA were administered locally in the nAc via reversed dialysis. Local administration of ethanol (300 mM) induced a significant elevation of extracellular dopamine as compared to Ringer control (two-way ANOVA with repeated measures time points 60–180 min; F (5, 44) = 7.8; p < 0.0001, Tukey’s *post hoc* analysis, Ringer vs. ethanol, p = 0.0005; Figure 3A). TPMPA did not influence dopamine on its own (Ringer vs. TPMPA 10 μM, p = 0.978; Ringer vs. TPMPA 100 μM, p = 0.941), but pre-treatment with either 10 μM or 100 μM TPMPA attenuated the ethanol-induced dopamine elevation (ethanol vs. TPMPA 10 μM + ethanol, p = 0.006 (Figure 3B); ethanol vs. TPMPA 100 μM + ethanol p = 0.004 (Figure 3C)), suggesting GABA-rho receptors to be involved in the ethanol-induced increase of dopamine. To verify the role of GlyRs in ethanol’s enhancement of the dopamine system, we locally administered the selective and competitive GlyR antagonist strychnine (20 μM) prior to local ethanol application. In line with our previous result (Molander and Söderpalm, 2005a), we were able to repeat that strychnine administration prevents the ethanol induced elevation of dopamine (two-way ANOVA with repeated measures time points 60–180 min; F _(3, 30)_ = 7.8; p = 0.0005, *post hoc* analysis, Ringer vs. ethanol p = 0.0024, Ringer vs. strychnine p = 0.963, Ringer vs. strychnine + ethanol p = 0.976, strychnine + ethanol vs. ethanol p = 0.0072) (Figure 3D).

### 3.3 Both glycine receptor and GABA-rho receptor blockade modulate the ethanol-induced increase of glycine and taurine

**FIGURE 4:**
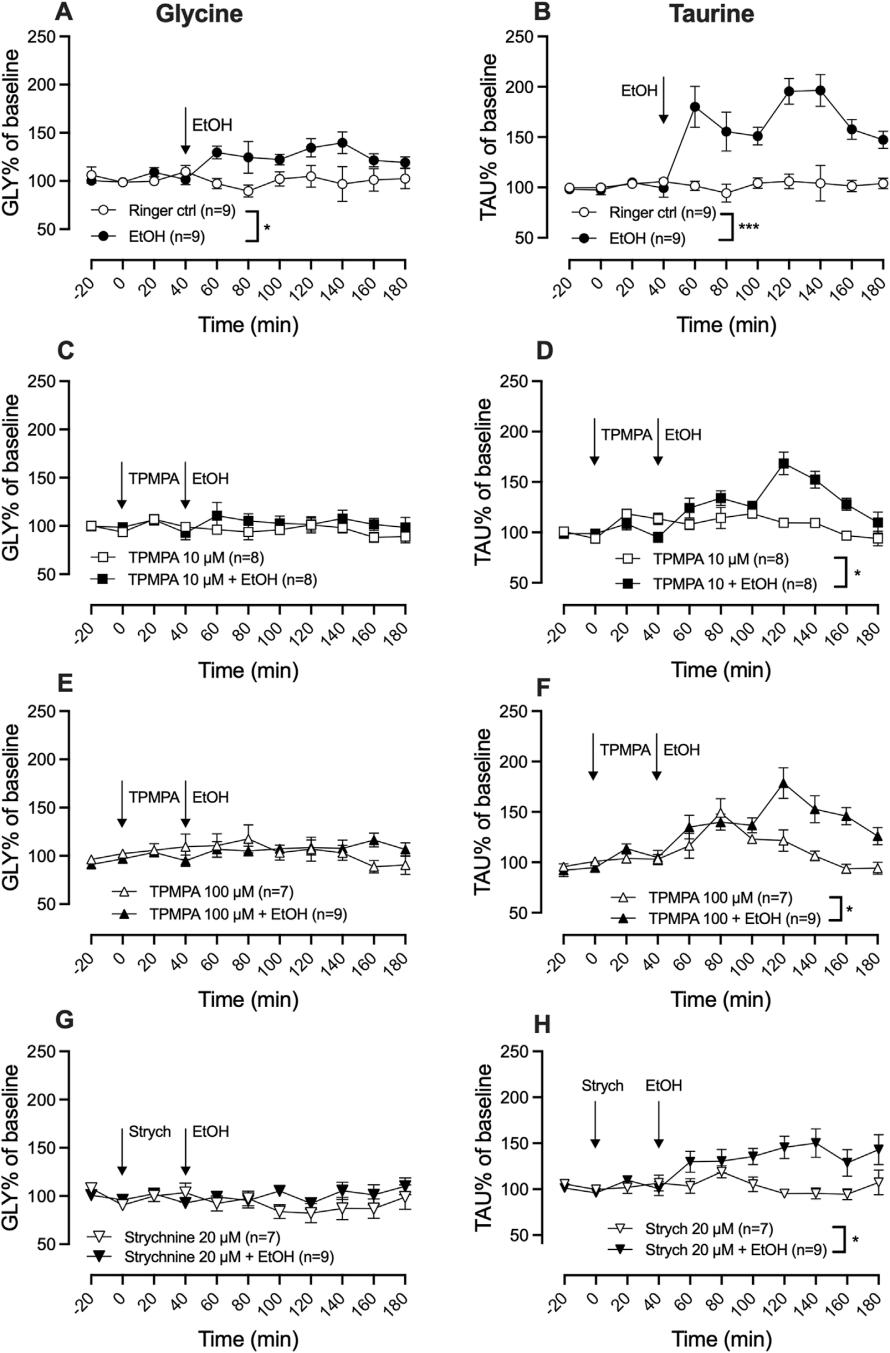
Blocking GABA-rho or glycine receptors attenuates ethanol-induced glycine and taurine elevation in the nucleus accumbens. Time-course graph of glycine **(A,C,E,G)** and taurine **(B,D,F,H)** levels as measured by *in vivo* microdialysis in freely moving Wistar rats, presented as percent of baseline following the local perfusion of Ringer’s solution and ethanol (EtOH) 300 mM **(A,B)**, TPMPA 10 μM alone and in combination with EtOH 300 mM **(C,D)**, TPMPA 100 μM alone and in combination with EtOH 300 mM **(E,F)**, strychnine (strych) 20 μM alone or in combination with EtOH **(G,H)**. Arrows indicate start of drug perfusion. Data are shown as means ± SEM. Statistical significance was determined by two-way repeated measures ANOVA followed by Tukey’s *post hoc* test, * denotes p < 0.05 and ***p < 0.001.

Activation of GlyRs is a prerequisite for ethanol-induced dopamine release and local administration of ethanol (300 mM) significantly increased extracellular levels of both glycine (two-way ANOVA with repeated measures time points 60–180 min; F (5, 44) = 3.31; p = 0.013, Tukey’s *post hoc* analysis, Ringer vs. ethanol, p = 0.040) (Figure 4A) and taurine (two-way ANOVA with repeated measures time points 60–180 min; F _(5, 44)_ = 17.62; p < 0.0001, *post hoc* analysis, Ringer vs. ethanol, p < 0.0001) (Figure 4) as compared to control.

To assess whether ethanol-induced elevation of glycine and/or taurine could be modulated by GABA-rho receptors, TPMPA (10 μM or 100 μM) was administered prior to the start of local ethanol-perfusion (300 mM). Pre-treatment with TPMPA did not significantly alter extracellular glycine levels as compared to control (Tukey’s *post hoc* analysis; Ringer vs. TPMPA 10 μM, p = 0.995; Ringer vs. TPMPA 100 μM, p = 0.998) but inhibited ethanol’s ability to increase glycine (TPMPA 10 µM vs. TPMPA 10 µM + ethanol, p = 0.902; TPMPA 100 µM vs. TPMPA 100 µM + ethanol, p = 0.990; Figures 4C,E). Perfusion of the GABA-rho antagonist did not influence extracellular levels of taurine alone (Tukey’s *post hoc* analysis; Ringer vs. TPMPA 10 μM, p = 0.993; Ringer vs. TPMPA 100 μM, p = 0.732). However, *post hoc* analysis found TPMPA to partially inhibit the ethanol-induced elevation of taurine (TPMPA 10 µM vs. TPMPA 10 µM + ethanol, p = 0.043; TPMPA 100 µM vs. TPMPA 100 µM + ethanol, p = 0.021; ethanol vs. TPMPA 10 µM + ethanol, p = 0.004 and ethanol vs. TPMPA 100 µM + ethanol, p = 0.068; Figures 4D,F). Similar to TPMPA, strychnine administration also inhibited the ethanol induced elevation of glycine (two-way ANOVA with repeated measures time points 60–180 min; F (3, 30) = 5.2; p = 0.0050, *post hoc* analysis, Ringer vs. ethanol p = 0.031, Ringer vs. strychnine p = 0.797, Ringer vs. strychnine + ethanol p = 0.997, strychnine + ethanol vs. ethanol p = 0.049; Figure 4G) and partially the ethanol induced elevation of taurine (two-way ANOVA with repeated measures time points 60–180 min; F _(3, 30)_ = 16.2; p < 0.0001, *post hoc* analysis, Ringer vs. ethanol p < 0.0001, Ringer vs. strychnine p = 0.999, Ringer vs. strychnine + ethanol p = 0.0152, strychnine vs. strychnine + ethanol p = 0.028; Figure 4H).

### 3.4 An artificial increase in glycine and taurine does not rescue ethanol-induced dopamine release

**FIGURE 5:**
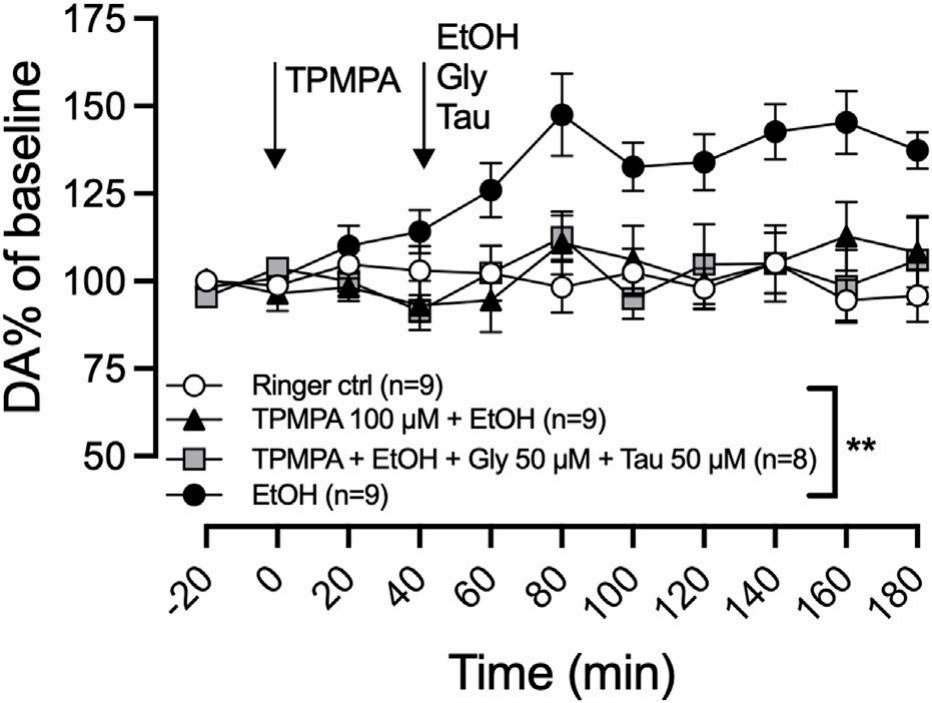
An artificial increase in glycine and taurine does not rescue ethanol-induced dopamine release. Time-course graph of extracellular levels of dopamine (DA) as measured by *in vivo* microdialysis in freely moving rats. Local administration, by means of reversed microdialysis in the nucleus accumbens, of Ringer’s solution, TPMPA 100 μM ethanol (EtOH) 300 mM, and in one group of rats, the addition of EtOH, glycine (Gly; 50 μM) and taurine (Tau; 50 μM) as indicated by the arrows. Data are shown as means ± SEM. Statistical significance was determined by two-way repeated measures ANOVA followed by Tukey’s *post hoc* test, ** denotes p < 0.01.

Considering the involvement of GlyRs in ethanol-induced dopamine release and the significant reduction of GlyR amino acid agonists following pretreatment with GABA-rho antagonist, we speculated that reduced GlyR signaling could underlie the effect of TPMPA. To test this hypothesis, we co-perfused a low level of glycine and taurine (50 µM of each amino acid via reversed microdialysis), which by themselves had no effect on basal dopamine. Co-perfusion of taurine has previously been demonstrated to restore ethanol-induced dopamine release during hypotonic conditions (Ericson et al., 2011). We administered TPMPA (100 µM) locally in the nAc 40 min before the addition of glycine (50 µM), taurine (50 µM), and ethanol (300 mM) in the perfusate. The addition of glycine and taurine did not restore the ethanol-induced dopamine elevation following GABA-rho blockade (two-way ANOVA with repeated measures time points 60–180 min; F (4, 37) = 5.9; p = 0.0009, *post hoc* analysis, Ringer vs. ethanol p = 0.0058, Ringer vs. TPMPA (100 µM)+ethanol p = 0.999, TPMPA (100 µM)+ethanol vs. TPMPA (100 µM)+ethanol + glycine + taurine) p = 0.999; Figure 5).

### 3.5 GABA-rho receptors are not involved in acamprosate-induced dopamine elevation

**FIGURE 6:**
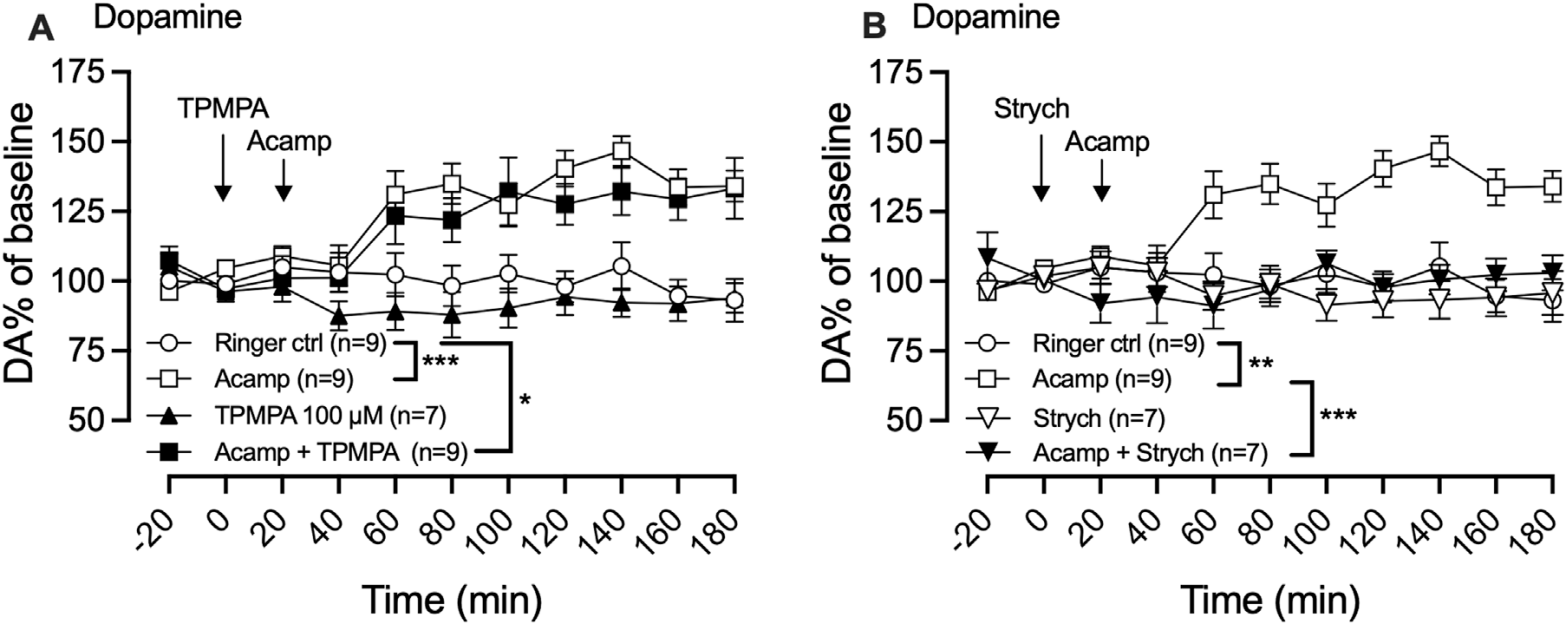
Acamprosate-induced dopamine elevation does not involve GABA-rho receptors. Time-course graph of extracellular levels of dopamine (DA) as measured by *in vivo* microdialysis in freely moving rats. Local administration, by means of reversed microdialysis in the nucleus accumbens, of Ringer’s solution, acamprosate (Acamp) 1 mM, TPMPA (100 μM), and the combination of acamprosate and TPMPA **(A)** and Ringer’s solution, acamprosate (Acamp) 1 mM, Strychnine (Strych; 20 μM), and the combination of acamprosate and strychnine **(B)**. Arrows indicate start of local drug perfusion. Data are shown as mean ± SEM. Statistical significance was determined by two-way repeated measures ANOVA followed by Tukey’s *post hoc* test, * denotes p < 0.05, **p < 0.01 and ***p < 0.001.

In a final set of experiments, we aimed to investigate the potential involvement of GABA-rho receptors in the acamprosate-induced increase in dopamine levels within the nAc. Local administration of acamprosate (1 mM) significantly increased dopamine levels in the nAc (two-way ANOVA with repeated measures time points 40–180 min; F (3, 30) = 10.15; p < 0.0001, *post hoc* analysis, Ringer vs. acamprosate p = 0.0004), an effect that was not affected by TPMPA 100 μM pre-treatment (Ringer vs. TPMPA p = 0.758, acamprosate vs. acamprosate + TPMPA p = 0.856, Ringer vs. acamprosate + TPMPA p = 0.023, Figure 6A), but fully blocked by strychnine (two-way ANOVA with repeated measures time points 40–180 min; F (3, 28) = 11.62; p < 0.0001, *post hoc* analysis, Ringer vs. acamprosate p = 0.0031, Ringer vs. strychnine p = 0.942, acamprosate vs. acamprosate + strychnine p < 0.001, Ringer vs. acamprosate + strychnine p = 0.999; Figure 6B).

## 4 Discussion

Recent studies have highlighted a role for GABA-rho receptors in ethanol-induced responses (Blednov et al., 2014; Yorgason et al., 2022). Considering the structural and functional similarity with GlyRs we hypothesized that the mechanisms associated with inhibition of GABA-rho receptors would be mimicked by GlyR antagonism. The data presented here demonstrate that GlyRs display a stronger influence over baseline dopamine levels and that a GlyR antagonist more robustly inhibits ethanol-induced dopamine release. In addition, while both TPMPA and strychnine reduce the ethanol-mediated release of GlyR agonists, only strychnine blocks dopamine release mediated by acamprosate. Thus, while GABA-rho receptors and GlyRs share some features, inhibition of GlyRs displays a more prominent effect on dopamine signaling and neurochemical responses to ethanol. Still, strychnine presents properties, e.g., deadly blockade of spinal GlyRs, that hinder its implementation as a possible intervention for AUD, while TPMPA appears to exert more ethanol-specific modulatory effects. Thus, GABA-rho receptors could be a promising target for future intervention strategies.

**FIGURE 7:**
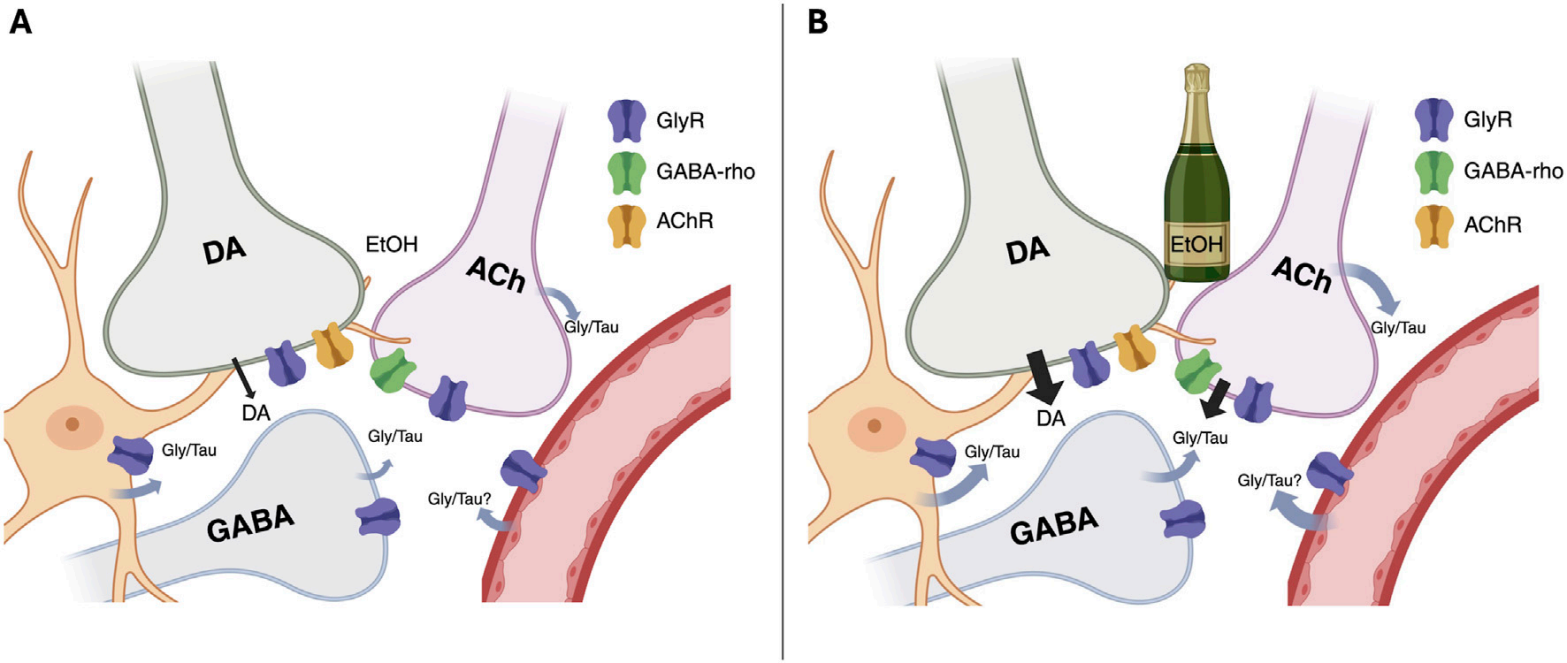
Simplified schematic illustrating the proposed roles of glycine receptors (GlyR) and GABA-rho receptors in regulating dopamine (DA) signaling under baseline conditions **(A)** and during ethanol exposure **(B)**. At baseline, DA tone in the nucleus accumbens is sustained by tonic GlyR activity on non-cholinergic interneurons, with little contribution from GABA-rho receptors. Ethanol elevates extracellular glycine and taurine, which activate GlyRs on cholinergic interneurons (CINs), enhancing acetylcholine (ACh) release and subsequent acetylcholine receptor (AChR)-mediated DA output. Full expression of this ethanol-induced DA elevation additionally requires GABA-rho receptors, as blockade of either receptor type reduces glycine/taurine signaling and prevents DA enhancement. Together, these findings suggest that ethanol recruits a “dual-key” mechanism involving both GlyR and GABA-rho signaling. The image was created using BioRender.

Local administration of the GlyR antagonist strychnine significantly reduced the microdialysate concentration of dopamine, indicating that nAc GlyRs are tonically active and play a key role in maintaining basal dopamine levels. This is in agreement with previous studies, demonstrating that strychnine produces a concentration-dependent decrease in accumbal dopamine concentrations which can be reversed by co-perfusion with glycine (Molander and Söderpalm, 2005a). TPMPA did not modulate extracellular dopamine levels, suggesting that GABA-rho receptors are not involved in maintaining basal dopamine levels in the nAc. This is further in line with an *in vitro* study, showing that TPMPA did not alter evoked dopamine release (Yorgason et al., 2022). The mechanism underlying GlyR-mediated regulation of dopamine levels has not been fully established, but it has been suggested that the GlyRs that are responsible for maintaining basal dopamine levels are not located on cholinergic interneurons (CINs), but rather involve other neurotransmitter systems (Loftén et al., 2023). Even though GABA-rho receptors may be expressed on a variety of neuronal cell types within the nAc, they appear to especially modulate CIN signaling (Yorgason et al., 2022). Thus, there appears to be a functional difference between glycine and GABA-rho receptors in the regulation of accumbal dopamine levels, which may be attributed to the distribution of expression. While GlyRs, putatively located on medium spiny neurons, appear to be critical for maintaining basal dopaminergic tone, GABA-rho receptors may play a more nuanced role, contributing to the dopaminergic response under conditions of ethanol exposure (Figure 7).

Ethanol increased accumbal dopamine levels, which is in agreement with previous studies (Ericson et al., 1998; Ericson et al., 2020; Imperato and Di Chiara, 1986). The increase in dopamine was fully blocked by the GlyR antagonist strychnine and significantly reduced by the GABA-rho receptor antagonist TPMPA. Accumbal dopamine signaling is tightly regulated by various neurotransmitter systems, with cholinergic transmission identified as a key regulator of dopamine release (Abrahao et al., 2017; Söderpalm and Ericson, 2013; Zhou et al., 2001). Endogenous acetylcholine derives mainly from local CIN and selective activation of these cells is sufficient to induce a dopamine increase, an effect mediated via nicotinic acetylcholine receptors located on dopamine terminals (Brimblecombe et al., 2018; Cachope et al., 2012; Threlfell et al., 2012). Accumbal CINs have also been shown to be involved in ethanol-induced dopamine release (Loftén et al., 2021; Loftén et al., 2023; Wadsworth et al., 2023). CINs are tonically active (Aosaki et al., 1995), and are regulated by glutamatergic inputs from the cortex and GABAergic projection neurons originating in the VTA (Al-Hasani et al., 2021; Brown et al., 2012). Since CIN may express both GlyRs and GABA-rho receptors (Sergeeva and Haas, 2001; Yorgason et al., 2022), it is possible that the mechanisms underlying the inhibitory effect of strychnine and TPMPA on ethanol-induced dopamine elevation involve modulation of CIN signaling. This is further supported by previous findings, demonstrating that cholinergic antagonists block the ethanol-induced dopamine increase and that selective depletion of CIN attenuates ethanol-induced dopamine elevation (Loftén et al., 2021).

Glycine receptors are important regulators of ethanol-induced dopamine elevation in rats (Jonsson et al., 2014; Molander and Söderpalm, 2005b). Glycine and taurine are endogenous agonists for the GlyR (Pan and Slaughter, 1995), and the data presented here demonstrate a robust increase in both of these amino acids in response to ethanol challenge. Changes in endogenous GlyR agonists could thus play a key role in regulating the acute rewarding properties of alcohol. In fact, the ethanol-induced rise in taurine levels has previously been shown to be a prerequisite for ethanol-induced dopamine release (Ericson et al., 2011). Importantly, both strychnine and TPMPA blunted the ethanol-induced release of glycine and taurine. As both receptors may be expressed by CIN (Sergeeva and Haas, 2001; Yorgason et al., 2022), and as depletion of accumbal CINs has been shown to reduce ethanol-induced levels of glycine and taurine (Loftén et al., 2023), these findings could be mediated through changes in cholinergic signaling. The role of CINs in regulating amino acid release needs however to be further evaluated. Postulating that the release of glycine and taurine is required for ethanol-induced dopamine release, we speculated that co-perfusion of these agonists could restore dopamine signaling during conditions where the GABA-rho receptor was blocked. However, TPMPA blocked ethanol-induced dopamine release also during these circumstances. Since glycine and taurine are also endogenous agonists for the GABA-rho receptor (Calvo and Miledi, 1995; Ochoa-de la Paz et al., 2008), it is possible that the inhibition of the receptor prevents downstream signaling. Thus, GABA-rho receptors may be important not only for inducing the release of glycine and taurine, but also for conveying downstream signaling mediated by these amino acids.

Acamprosate is a pharmacological treatment for AUD approved by the United States Food and Drug Administration (FDA) and the European Medicines Agency (EMA). The mechanism of action is debated where some suggest modulation of N-Metyl-D-aspartate (NMDA) receptors, stabilizing excitatory and inhibitory neurotransmission, whilst others suggest interaction with accumbal GlyRs or that calcium and not homotaurine may underlie the effects of acamprosate (Ademar et al., 2022; Ademar et al., 2023; Al Qatari et al., 1998; Chau et al., 2010a; Chau et al., 2018; Spanagel et al., 2014). The interaction with GlyRs is suggested to mediate the acamprosate-induced dopamine increase, a pharmacological effect proposed to be important in the decreased ethanol consumption seen following treatment. Therefore, we wanted to investigate if part of acamprosate’s dopamine-releasing effect is mediated via GABA-rho receptors as this information could have important treatment implications. Supporting previous studies, we found that strychnine blocked dopamine release elicited by acamprosate. However, TPMPA did not attenuate acamprosate-induced dopamine increase, suggesting that GABA-rho receptors are not involved in acamprosate’s effect on dopamine. However, it should be noted that the present study was performed exclusively in male rats. Future studies should include both sexes to ensure broader applicability of the findings and to investigate potential sex differences.

In summary, our *in vivo* findings demonstrate that GABA-rho receptors in the nAc play an important role in mediating ethanol-induced elevation of dopamine levels and can influence ethanol-induced release of the GlyR agonists glycine and taurine. However, the GABA-rho receptor does not appear to contribute to the maintenance of basal dopamine levels in the nAc or to the dopamine-elevating effects of acamprosate. Although GlyR antagonism had a more prominent effect on dopamine signaling, the observation that GABA-rho receptors neither affect basal dopamine neurotransmission nor non-ethanol induced stimulation but appear to exert an ethanol-specific modulatory effect could make it a promising target for pharmacological interventions. Overall, these results highlight a selective involvement of GABA-rho receptors in ethanol-related neurochemical responses, providing new insights into the neurobiological mechanisms underlying ethanol’s effects on the reward system.

## Data Availability

The raw data supporting the conclusions of this article will be made available by the authors, without undue reservation.
